# Prognostic utility of the chest computed tomography severity score for the requirement of mechanical ventilation and mortality in hospitalized patients with COVID-19

**DOI:** 10.1016/j.heliyon.2023.e16020

**Published:** 2023-05-02

**Authors:** Yukiyoshi Kimura, Cesar N. Cristancho-Rojas, Yumi Kimura-Sandoval, Ramiro Tapia-Sosa, Lorena Guerrero-Torres, Mariana Licano-Zubiate, Monica Chapa-Ibargüengoitia

**Affiliations:** a Department of Radiology, Instituto Nacional de Ciencias Medicas y Nutricion Salvador Zubiran, Vasco de Quiroga 15, Belisario Domínguez Secc 16, Tlalpan, 14080, Mexico City, Mexico; b Radiology Department, CT Scanner Group, Puebla 228, Roma Norte, 06700, Mexico City, Mexico; c Department of Gastroenterology, Instituto Nacional de Ciencias Medicas y Nutricion Salvador Zubiran, Vasco de Quiroga 15, Belisario Domínguez Secc 16, Tlalpan, 14080, Mexico City, Mexico; d Department of Infectious Diseases, Instituto Nacional de Ciencias Medicas y Nutricion Salvador Zubiran, Vasco de Quiroga 15, Belisario Domínguez Secc 16, Tlalpan, 14080, Mexico City, Mexico

**Keywords:** COVID-19, Diagnostic imaging, Chest, Multidetector computed tomography

## Abstract

**Purpose:**

To correlate the chest computed tomography severity score (CT-SS) with the need for mechanical ventilation and mortality in hospitalized patients with COVID-19.

**Materials and methods:**

The chest CT images of 224 inpatients with COVID-19, confirmed by reverse transcriptase-polymerase chain reaction (RT-PCR), were retrospectively reviewed from April 1 to 25, 2020, in a tertiary health care center. We calculated the CT-SS (dividing each lung into 20 segments and assigning scores of 0, 1, and 2 due to opacification involving 0%, <50%, and ≥50% of each region for a global range of 0–40 points, including both lungs), and collected clinical data. The receiver operating characteristic curve and Youden Index analysis was performed to calculate the CT-SS threshold and accuracy for classification for risk of mortality or MV requirement.

**Results:**

136 men and 88 women were recruited, with an age range of 23–91 years and a mean of 50.17 years; 79 met the MV criteria, and 53 were nonsurvivors. The optimal threshold was >27.5 points for mortality (area under ROC curve >0.96), with a sensitivity of 93% and specificity of 87%, and >25.5 points for the need for MV (area under ROC curve >0.94), with a sensitivity of 90% and specificity of 89%. The Kaplan-Meier curves show a significant difference in mortality by the CT-SS threshold (Log Rank p < 0.001).

**Conclusions:**

In our cohort of hospitalized patients with COVID-19, the CT-SS accurately discriminates the need for MV and mortality risk. In conjunction with clinical status and laboratory data, the CT-SS may be a useful imaging tool that could be included in establishing a prognosis for this population.

## 1 Introduction

The current SARS-CoV-2 pandemic has caused global uncertainty at an unprecedented scale, having infected more people and taken more lives compared with the combined effects of SARS and MERS [[1], [2], [3]]. It has spread over a relatively short time and has caused healthcare services saturation, with exceeded hospital and intensive care unit bed capacity in some countries worldwide [4].

The definitive diagnosis of COVID-19 relies on real-time reverse transcriptase-polymerase chain reaction (RT-PCR) [5, 6, 7], but this has a high false-negative rate [8]. In several situations, the availability and processing time of the test makes the diagnosis inopportune. In these situations, radiology plays an important role. Noncontrast chest computed tomography (CT) has a higher pooled sensitivity (93%) [5] for COVID-19 compared with that of RT-PCR (71%–98%) [5] but lower specificity [9]. It is a valuable additional tool for identifying patients with moderate to severe COVID-19 and those with worsening respiratory status [10, 11].

Compared with chest radiography, thin-section chest CT has a higher sensitivity and can detect subtle lung parenchyma changes in the early stages of the disease [7]. According to the Fleischer Society consensus statement [10], thorax imaging is indicated in patients with mild features of COVID-19 and have risk factors for progressive or worsening respiratory status, as well as for medical triage of patients with moderate to severe clinical features in resource-constrained environments. In patients in such clinical scenarios, chest CT allows the assessment of the virus' pulmonary damage [5].

To quickly and objectively identify patients with severe diseases, especially in institutions with limited healthcare resources, Yang et al. [7] developed a practical and easy semiquantitative scoring method. The CT severity score (CT-SS) is based on the amount of lung opacification in 20 regions. A higher CT-SS has been observed in patients with severe disease than in mild cases. A CT-SS threshold of 19.5 was reported to have 83.3% sensitivity and 94% specificity in identifying patients with severe COVID-19 [7].

However, no objective imaging measurement enables the prediction of the need for mechanical ventilation (MV) and mortality among hospitalized patients with COVID-19 among different populations (i.e., Hispanic). This study intended to evaluate the discriminating accuracy of a practical CT-SS score for the need for MV and mortality in hospitalized COVID-19 patients in a tertiary referral center in Mexico City.

## 2 Materials and methods

### 2.1 Patients and groups

This observational retrospective study protocol followed the Declaration of Helsinki's ethical principles. The research and ethics committees of the National Institute of Nutrition and Medical Sciences approved this study (Reference number: 3414). Due to the retrolective methodology, the Informed consent was waived, although all data and imaging information was anonymized.

We retrospectively reviewed the chest CT examinations of 224 consecutive inpatients (in order of hospitalization) with COVID-19 confirmed by RT-PCR from April 1 to 25, 2020. According to our hospital protocol at that time, all hospitalized patients with suspected COVID-19 pneumonia underwent non-contrast chest CT. Hospitalized patients with clinical and radiologic manifestations and a negative RT-PCR result (43 patients) were excluded. The patients were classified according to outcomes as survivors or nonsurvivors and whether they met MV's criteria. The criteria for MV were: 1) no improvement in respiratory distress despite the use of a nonrebreather mask with high-flow oxygen (10–15 L/min), with a respiratory rate higher than 30 per minute, the partial pressure of oxygen (PaO_2_) < 60 mmHg, and a PaO_2_/fraction of inspired oxygen (FiO_2_) < 150 with respiratory distress while on noninvasive respiratory support; and 2) any patient with PaO_2_/FiO_2_ < 100.

### 2.2 Chest CT severity score calculation

**Fig. 1:**
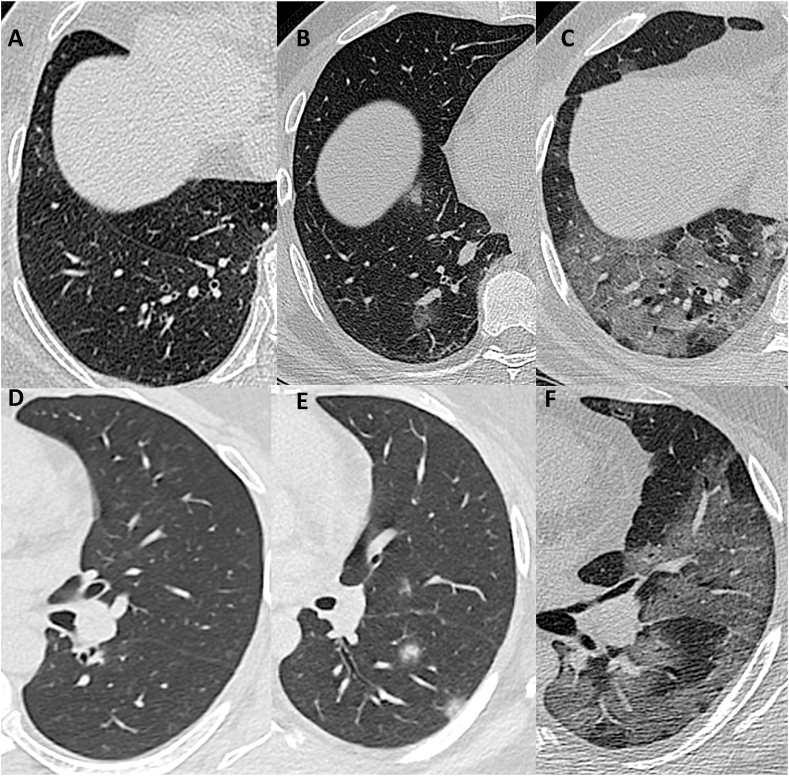
Representative axial non-contrast chest CT images from different patients to show the calculation of the CT-SS in the right posterobasal segment (S10) (A–C) and left superior segment (S6) (D–F). In images A and D, the absence of ground glass opacities or consolidation correspond to 0 points. In images B and E, less than 50% compromised segment corresponds to 1 point. In images C and F, diffuse ground glass opacities in more than 50% of the studied segments correspond to 2 points.

As proposed by Yang et al. [7], the CT-SS for the assessment of COVID-19 entails the division of the lungs into 20 regions; specifically, the apicoposterior segment of the left upper lobe is divided into the apical and posterior regions, and the anteromedial basal segment of the left lower lobe is divided into medial basal and anterior basal regions. For each patient, CT-SS of 0, 1, and 2 were assigned for parenchymal opacification involving 0%, <50%, and ≥50% of each region, respectively (global range of 0–40 points, including both lungs) as is illustrated in Fig. 1(A–F). Only the initial CT scan was reviewed in patients with multiple chest CT scans (28 patients).

The CT scans, and CT-SS were independently reviewed and calculated for outcomes correlation in a standard diagnostic system workstation by a general radiologist with more than ten years of experience and blinded to the clinical and biochemical data. We assessed the interobserver agreement by including the scores of 25 CTs from two other independent and blinded imaging reviewers: a general radiologist with five years of experience and a radiologist in training who made determinations on his own and had recent experience in calculating the CT-SS of other 25 scans that were not included in the present study, training under supervision the radiologist with more than ten years of experience.

### 2.3 Chest CT scans

Chest CT scans were performed on a 64-detector CT scanner (Revolution EVO, General Electric). All CT examinations were performed with the patient in a supine position, with images acquired from the lung apex to the adrenal glands during a single inspiratory breath. The CT scan parameters were as follows: 120 kV, 350 mAs; rotation time of 0.4 s; the pitch of 1.53:1 (mm/rot); intersection space of 5 mm; multiplanar reconstructions with a slice thickness of 1.5 mm; and a sharp convolution kernel. Thin-section CT scans were read at a window width of 1000–2000 HU and level of −700 to −500.

### 2.4 Statistical analysis

A comparative analysis between the survivors and nonsurvivors was performed along with the descriptive analysis (dispersion and central tendency statistics). The hypotheses for comparing numerical variables were tested using Student's t-test, and categorical variables were compared using the chi-square test. Due to a lack of Gaussian distribution of CT-SS within mortality and MV groups (Shapiro–Wilk test *p* = 0.03), we assess these with a Mann–Whitney *U* test. The analysis was performed using STATA SE 14.1 software and SPSS software package version 20, considering a significant two-tailed *p*-value of <0.05.

We used the intraclass correlation coefficient (ICC) to estimate the interrater variability and absolute agreement on the CT-SS among the three observers. We tested the diagnostic yield of the global CT-SS using receiver operating characteristic (ROC) curve analysis to describe the sensitivity, specificity, positive predictive value (PPV), negative predictive value (NVP), positive likelihood ratio (LR+), negative likelihood ratio (LR−), odds ratio (OR), and area under the ROC curve (AUC) with 95% confidence interval (CI). Days of hospitalization and relevant time intervals between the onset of symptoms, RT-PCR realization, CT acquisition, and the outcomes were reported. Furthermore, we built a Kaplan-Meier curve for mortality regarding the CT-SS for visual assessment and Log Rank regression.

We explored the classification threshold using the Youden index, which was the optimal maximum difference between the true and false positive ratios. Notably, the original identification study reported a predetermined threshold of 19.5 [7].

## 3 Results

### 3.1 Clinical and demographic variables associated with mortality

**Fig. 2:**
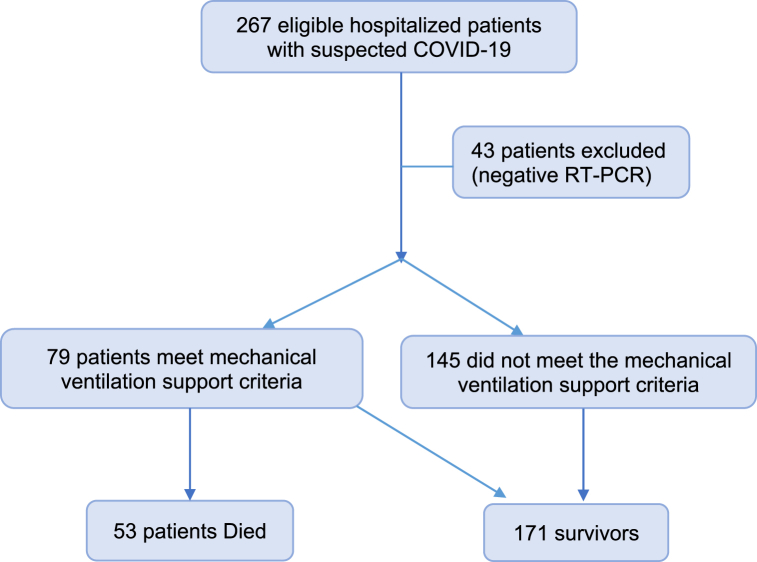
Flow diagram of the study participants.

The 224 patients included (Fig. 2) had a mean age of 50.17 years (SD, 13.85) and an age range of 23–91 years; 60.7% (136/224) were men, and 39.29% (88/224) were women. Mortality was observed in 23% (53/224) of the patients and was significantly higher in those with older age than those with younger age (57 ± 14 years vs. 48 ± 13 years, *p* < 0.001); in men than in women (75.5% vs. 56.1%, *p* = 0.012); in those who met the criteria for MV than in those who did not (96.2% vs. 16.4%, *p* < 0.001); in those with diabetes than in those without diabetes (38.5% vs. 21.1%, *p* = 0.01); and in those with organ transplant than in those without organ transplant (11.3% vs. 3.5%, *p* = 0.027). Table 1 compares the demographic and clinical features between survivors and nonsurvivors.

**Table 1:** Demographic data and comparison between survivors and nonsurvivors^a^.

	Survivors (n = 171)	Nonsurvivors (n = 53)	*p*-value
Age, years mean (SD)	48 (13)	57 (14)	<0.001
Male	96 (56.1)	40 (75.5)	0.012
Patients met the criteria for MV	28 (16.4)	51 (96.2)	<0.001
Patients with access to MV^b^	26 (15.2)	41 (77.4)	<0.001
Length of stay, days mean (SD)	10.24 (8.03)	10.36 (9.2)	0.9
The time between onset and CT, days mean (SD)	8.5 (5.01)	7.67 (3.72)	0.25
**Symptoms upon onset**			
Fever	150 (87.7)	48 (90.6)	0.57
Cough	132 (77.2)	46 (86.8)	0.13
Diarrhea	36 (21.1)	8 (15.1)	0.34
**Comorbidities**			
Diabetes	36 (21.1)	20 (38.5)	0.01
Hypertension	36 (21.1)	18 (34.0)	0.055
Smoker	3 (1.8)	3 (5.7)	0.12
Asthma	2 (1.2)	0 (0.0)	0.42
Obesity	27 (15.8)	14 (26.4)	0.08
Immunosuppression	6 (3.5)	2 (3.8)	0.92
Cardiovascular disease	2 (1.2)	1 (1.9)	0.69
Organ transplant	6 (3.5)	6 (11.3)	0.027
**CT findings**			
Right lung CT-SS, mean (SD)	10.38 (3.7)	16.79 (2.55)	<0.001^c^
Left lung CT-SS, mean (SD)	10.05 (3.64)	16.88 (2.17)	<0.001^c^
Both lungs CT-SS, mean (SD)	20.27 (6,94)	30.68 (4.21)	<0.001^c^
Pleural effusion	3 (1.8)	0 (0.0)	0.3
Nodes	8 (4.7)	3 (5.7)	0.7

SD: standard deviation, MV: mechanical ventilation, CT-SS: computed tomography severity score.

a Data are presented as n (%) unless specified.

b Patients without access to MV because of limited resources were not included.

c Assess with Mann–Whitney *U* test.

**Fig. 3:**
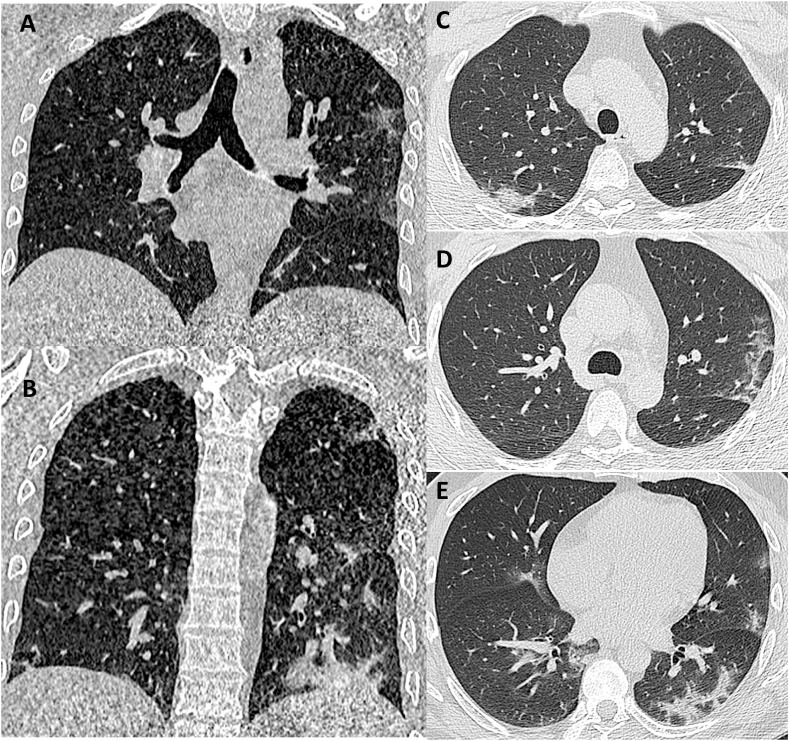
Noncontrast chest CT images of a 43-year-old man. Coronal reformatted (A and B), and axial (C–E) images show bilateral ground glass opacities in multiple lung segments and a CT-SS of 12.

**Fig. 4:**
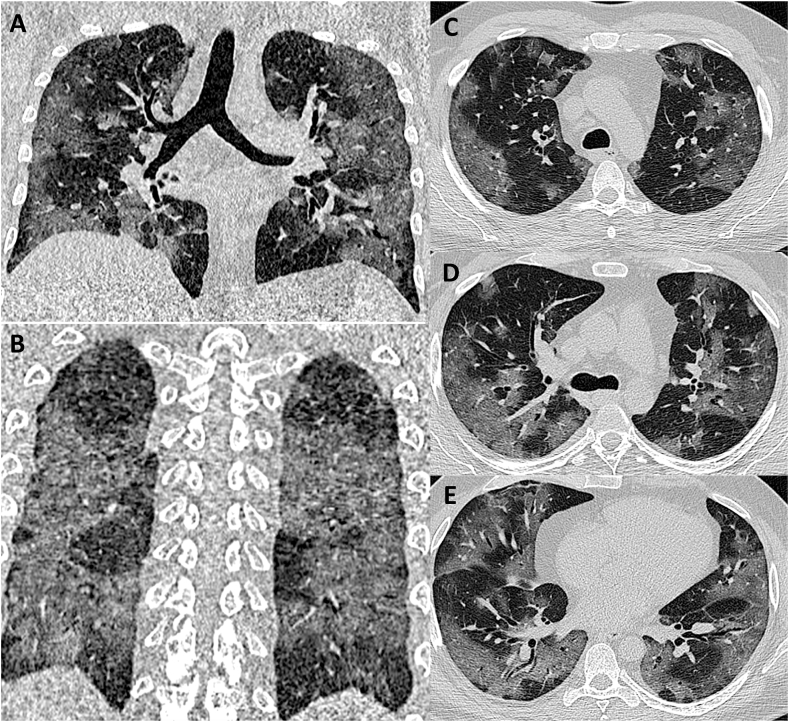
Noncontrast chest CT images of a 44-year-old man. Coronal reformatted (A and B), and axial (C–E) images show multiple ground glass opacities in multiple lung segments and a CT-SS of 32.

**Fig. 5:**
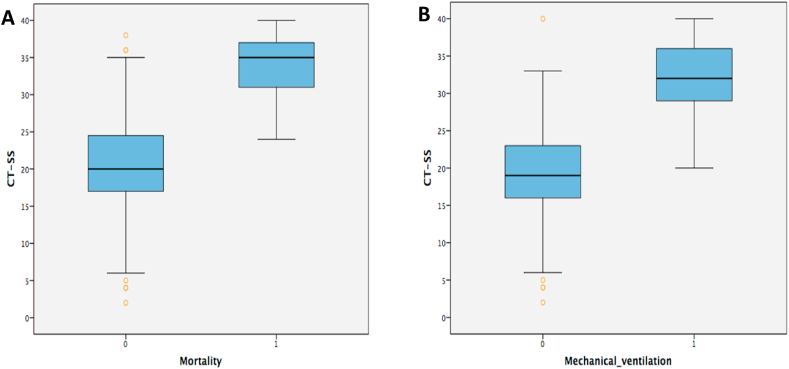
Box plots of the CT-SS in the mortality (A) and mechanical ventilation (B) groups *.

Compared with the survivors, the nonsurvivors had significantly higher CT-SS for the right lung (16.79 ± 2.55 vs. 10.38 ± 3.7, *p* < 0.001); CT-SS for the left lung (16.88 ± 2.17 vs. 10.05 ± 3.64, *p* < 0.001) [Fig. 3(A–F), Fig. 4(A–E)]. For both groups of mortality and meeting the MV criteria, the CT-SS (including both lungs) showed significant differences (p < 0.0001) [Table 2 and Fig. 5(A and B)].

**Table 2:** Comparison of CT-SS according to mortality and need for mechanical ventilation.

		CT-SS	
Outcome	(n)	Median (IQR)	Mann–Whitney (*p*)
Mechanical ventilation	No (145)	19 (16–23)	>0.0001
	Yes (79)	32 (29–36)	
Mortality	No (171)	20 (17–25)	>0.0001
	Yes (53)	35 (31–37)	

CT-SS: computed tomography severity score, IQR: interquartile range.

The time elapsed between the onset of symptoms to hospitalization, RT-PCR, and CT was: 8.11 ( ± 4.243), 8.33 ( ± 4.749) days, respectively, and 0.5 ( ± 2.567), 0.65 ( ± 1.475), and 2.21 ( ± 2.665), days between hospitalization to RT-PCR, CT, and MV. The time interval between RT-PCR and CT was 0.82 days ( ± 2.47) (Table 3).

**Table 3:** Time intervals between Onset of Symptoms, CT, RT-PCR, Hospitalization, and outcomes.

(Length in days)	Mean (SD)	Median (Percentile 5–95)	Minimum	Maximum
Symptoms to Hospitalization	8.11 ( ± 4.243)	7 (3–14.75)	0	31
Symptoms to RT-PCR	8.09 ( ± 4.568)	7 (3–16.75)	0	31
Symptoms to CT	8.33 ( ± 4.749)	7.5 (3–16.5)	1	32
RT-PCR to CT	0.82 ( ± 2.47)	0 (0–4)	0	25
Length of hospitalization	10.34 ( ± 8.95)	7.5 (3–33)	1	50
Hospitalization to RT-PCR	0.5 ( ± 2.567)	0 (0–1)	0	32
Hospitalization to CT	0.65 ( ± 1.475)	0 (0–4)	0	10
Hospitalization to MV (n = 79)	2.21 ( ± 2.665)	1 (0–8.1)	0	12

SD: standard deviation, MV: mechanical ventilation, SD: standard deviation, RT-PCR: real-time reverse transcriptase-polymerase chain reaction.

### 3.2 Diagnostic yield and a proposed threshold for CT-SS

**Fig. 6:**
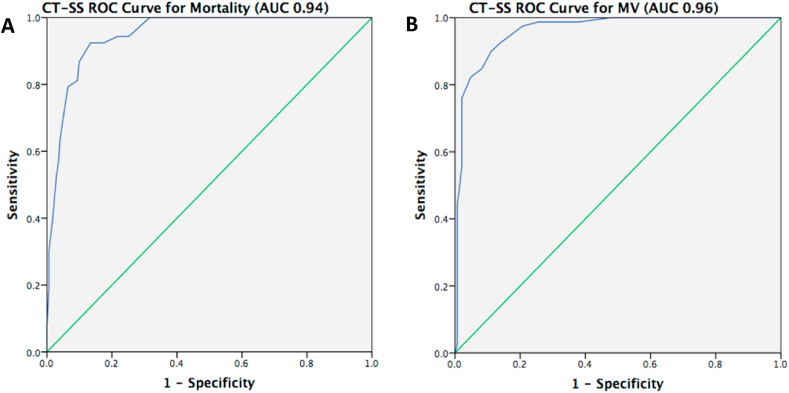
The area under the receiver operating characteristic curve of semiquantitative CT-SS for mortality (A) and MV requirement (B).

We plotted the ROC curves and found excellent AUCs for MV (0.96, 95% CI 0.939–0.985) and mortality (0.95, 95% CI 0.922–0.977) [Fig. 6(A and B)]. Using the Youden score, we found that the classification thresholds for CT-SS were >25.5 points for MV (OR 72, 95% CI 29.5–173.2) and >27.5 for mortality (OR 78.8, 95% CI 26.8–228.9). Besides, we validated the performance of the predetermined threshold of CT-SS >19.5. The details of the diagnostic accuracy of CT-SS are outlined in Table 4. We found an ICC for CT-SS (n = 25) of 0.97 (95% CI 0.95–0.99, *p* < 0.001).

**Table 4:** Diagnostic yield of the CT-SS for two different outcomes.

Variable (Prevalence)	Mechanical ventilation^a^ (35.3%)		Mortality (23.7%)	
Classification Threshold	**Severity score>25.5**	**Severity score>19.5**^b^	**Severity score>27.5**	**Severity score>19.5**^b^
AUC (95% CI)	0.894 (0.84–0.93)	0.762 (0.72–0.80)	0.895 (0.84–0.93)	0.722 (0.68–0.76)
Youden index	0.788	–	0.79	–
SEN (95% CI)	89.9 (81–95.5)	100 (95.4–100)	92.5 (81.8–97.9)	100 (93.3–100)
SPE (95% CI)	89 (82.7–93.6)	52.4 (44–60.8)	86.5 (80.5–91.3)	44.4 (36.9–52.2)
PPV (95% CI)	81.6 (71.9–89.1)	53.4 (45–61.6)	68.1 (56.0–78.6)	35.8 (28.1–44.1)
NPV (95% CI)	94.2 (88.8–97.4)	100 (95.3–100)	97.4 (93.4–99.3)	100 (95.3–100)
LR+ (95% CI)	8.14 (5.1–13.01)	2.1 (1.77–2.49)	6.87 (4.66–10.13)	1.80 (1.57–2.06)
LR− (95% CI)	0.11 (0.06–0.22)	0.0	0.09 (0.03–0.22)	0.0
OR (95% CI)	71.55 (29.48–173.21)	22.48	78.8 (26.8–228.9)	10.96

CT-SS: computed tomography severity score, AUC: area under the ROC curve, CI: confidence interval.

SEN: Sensitivity, SPE: specificity, PPV: positive predictive value, NPV: negative predictive value, LR: likelihood ratio, OR: odds ratio.

a Including all patients who met the criteria for mechanical ventilation.

b Threshold proposed by Yang et al. [7] for identifying patients with severe disease.

**Fig. 7:**
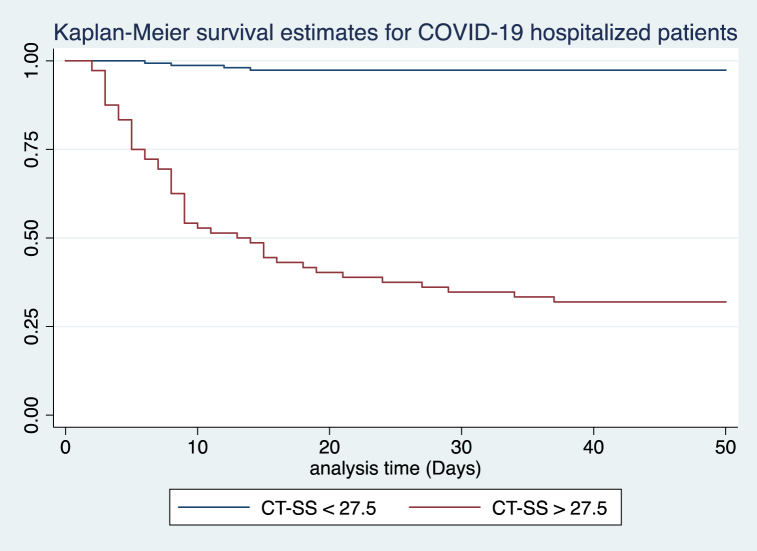
Kaplan-Meier survival graph comparing the CT-SS groups: computed tomography severity score (CT-SS) threshold.

The Kaplan-Meier curves show a significant difference (Log Rank p < 0.001) in mortality between groups separated by the proposed CT-SS threshold during 50 days of the observation period (Fig. 7); the median days of survival in the group with CT-SS > 27.5 was calculated at 22.9 (95% CI 18.4–27.5).

## 4 Discussion

We reported a study on the CT-SS classification accuracy, a semiquantitative imaging tool for the clinical outcomes of the requirement for MV and mortality in hospitalized patients with COVID-19, showing excellent AUCs of 0.94 and 0.96, respectively. Moreover, we found that the proposed CT-SS thresholds of >25.5 points for MV and >27.5 points for mortality had excellent classification performance, with respective sensitivities and specificities of 89.9% and 89% for MV, and 92.5% and 86.5% for mortality, plus robust likelihood ratios.

Several epidemiological studies reported variable rates of invasive MV [12], ranging from 2.3% [12] to 33.1% [13] among hospitalized patients. In our study, 35% of patients met the criteria for MV, and 29% had access to it. The higher mortality rate in this retrospective study than in previous reports [12] reflected the specific status of a cohort of hospitalized patients in a referral center. The mortality rate reported in our study (24%) was similar to the 21% mortality rate reported by Richardson et al. among hospitalized patients with COVID-19 [14]. Even though the OR might overestimate the risk ratio, it has been shown stability among study designs. In our population, the OR was a significant parameter in estimating the mortality and MV risk based on the CT-SS. Determining the need for MV will ultimately be clinical; however, a CT-SS >25.5 points may classify patients at high risk for respiratory deterioration and mortality.

Consistent with Yang et al. [7], we revealed an excellent agreement among readers, suggesting that CT-SS may be highly reproducible in radiology departments and diagnostic centers. However, our study did not include an expert on chest radiology. Although this could be regarded as a limitation, we believed it was our study's strength because an expert chest radiologist is only sometimes present in the interpretation room. The interpretation of chest CT using the CT-SS can be carried out even by radiologists in training. Additionally, a semiquantitative score could be used for patient follow-up and objectively assessing changes.

Furthermore, we validated the predetermined threshold of >19.5 in our cohort and found near-perfect sensitivities (100%) for MV and mortality but low specificities of 52.4% and 44.4%, respectively. Moreover, the AUCs for the predetermined cutoff point of 0.76 for MV and 0.72 for mortality were substantially less than those found for our proposed thresholds (>0.89), as well as OR and likelihood ratios.

Yuan et al. [15] retrospectively studied 27 consecutive patients and proposed a different severity score ranging from 0 to 72 and a threshold of 24.5 as a mortality predictor (85% sensitivity and 86% specificity). In a multicenter prospective study [16], the CT-SS was suggested for the triage and management decision of COVID-19 patients since it showed a significant association with the odds of hospital admission, ICU admission, and 30-day mortality. Also, Salvatore et al. found that CT visual score and software-based quantification of parenchymal involvement were independent predictors of outcomes in patients with COVID-19 pneumonia, proving that CT-SS identifies patients who need more aggressive treatment [17]. To our knowledge, no clinical trial has compared the different available scores.

In a systematic review and meta-analysis pooling 7106 patients, Zakariaee et al. found that the odds of mortality for COVID-19 patients could be accurately predicted using an optimal CT-SS cutoff in visual scores and that higher CT-SS correlates with higher odds of mortality [18]. Another recent meta-analysis showed that higher CT-SS strongly correlates with lung fibrosis [19].

This study had the limitation of having a retrospective cohort design in a single tertiary referral public institution. We only admitted hospitalized patients, and outpatients were excluded. Another limitation was that the subjects needed to be followed up after discharge, hindering information of mortality outcomes after hospital discharge. Multicenter studies on prospective cohorts are necessary for external validation.

In conclusion, in our cohort of hospitalized patients with COVID-19, the CT-SS accurately discriminates the need for MV and mortality. In conjunction with clinical status and biochemical data, the CT-SS may be a useful imaging tool that could be included in these patients' prognostication.

## Author contribution statement

Yukiyoshi Kimura, M.D.; Cesar Nicolas Cristancho-Rojas, M.D., M.Sc.: Conceived and designed the experiments; Performed the experiments; Analyzed and interpreted the data; Contributed reagents, materials, analysis tools or data; Wrote the paper.

Yumi Kimura-Sandoval; Ramiro Tapia-Sosa; Lorena Guerrero-Torres; Mariana Licano-Zubiate; Monica Chapa-Ibargüengoitia; Yukiyosi Kimura: Analyzed and interpreted the data; Contributed reagents, materials, analysis tools or data; Wrote the paper.

## Data availability statement

Data will be made available on request.

## Declaration of competing interest

The authors declare that they have no known competing financial interests or personal relationships that could have appeared to influence the work reported in this paper.

## Abbreviations

CT: Computed Tomography, CT-SS: Chest CT Severity Score, RT-PCR: reverse transcriptase-polymerase chain reaction, MV: Mechanical Ventilation, ICC: intraclass correlation coefficient, ROC: receiver operating characteristic, AUC: area under the ROC curve, PPV: positive predictive value, NPV: negative predictive value, LR: likelihood ratio, OR: odds ratio, CI: Confidence Interval.

## References

[1] Zhou P., Yang X.-L., Wang X.-G., Hu B., Zhang L., Zhang W. (2020 Jan 23). Discovery of a novel coronavirus associated with the recent pneumonia outbreak in humans and its potential bat origin. Nature [Internet].

[2] Drosten C., Günther S., Preiser W., van der Werf S., Brodt H.-R., Becker S. (2003 May 15). Identification of a novel coronavirus in patients with severe acute respiratory syndrome. N. Engl. J. Med [Internet].

[3] Peiris J.S.M., Lai S.T., Poon L.L.M., Guan Y., Yam L.Y.C., Lim W. (2003 Apr 19). Coronavirus as a possible cause of severe acute respiratory syndrome. Lancet.

[4] Yan Y., Shin W.I., Pang Y.X., Meng Y., Lai J., You C. (2020).

[5] Salameh J.P., Leeflang M.M.G., Hooft L., Islam N., McGrath T.A., van der Pol C.B. (2020 Sep 30).

[6] Huang C., Wang Y., Li X., Ren L., Zhao J., Hu Y. (2020 Feb 15). Clinical features of patients infected with 2019 novel coronavirus in Wuhan, China. Lancet.

[7] Yang R., Li X., Liu H., Zhen Y., Zhang X., Xiong Q. (2020). Chest CT severity score: an imaging tool for assessing severe COVID-19. Radiol Cardiothorac Imaging [Internet].

[8] Kucirka L.M., Lauer S.A., Laeyendecker O., Boon D., Lessler J. (2020 May 13). Variation in false-negative rate of reverse transcriptase polymerase chain reaction–based SARS-CoV-2 tests by time since exposure. Ann. Intern. Med..

[9] Fang Y., Zhang H., Xie J., Lin M., Ying L., Pang P. (2020). Sensitivity of chest CT for COVID-19: comparison to RT-PCR. Radiology [Internet].

[10] Rubin G.D., Ryerson C.J., Haramati L.B., Sverzellati N., Kanne J.P., Raoof S. (2020 Jul 7). The role of chest imaging in patient management during the COVID-19 pandemic: a multinational consensus statement from the fleischner society. Radiology [Internet].

[11] Simpson S., Kay F.U., Abbara S., Bhalla S., Chung J.H., Chung M. (2020). Radiological society of north America expert consensus statement on reporting chest CT findings related to COVID-19. Endorsed by the Society of Thoracic Radiology, the American College of Radiology, and RSNA. Radiol Cardiothorac Imaging [Internet].

[12] Guan W., Ni Z., Hu Y., Liang W., Ou C., He J. (2020 Apr 30). Clinical characteristics of coronavirus disease 2019 in China. N Engl J Med [Internet].

[13] Goyal P., Choi J.J., Pinheiro L.C., Schenck E.J., Chen R., Jabri A. (2020). Clinical characteristics of covid-19 in New York city. N Engl J Med [Internet].

[14] Richardson S., Hirsch J.S., Narasimhan M., Crawford J.M., McGinn T., Davidson K.W. (2020 May 26). Resenting characteristics, comorbidities, and outcomes among 5700 patients hospitalized with COVID-19 in the New York city area. JAMA [Internet].

[15] Yuan M., Yin W., Tao Z., Tan W., Hu Y., Schildgen O. (2020). Association of Radiologic Findings with Mortality of Patients Infected with 2019 Novel Coronavirus in Wuhan, China.

[16] Lieveld A.W.E., Azijli K., Teunissen B.P., van Haaften R.M., Kootte R.S., van den Berk I.A.H., van der Horst S.F.B., de Gans C., van de Ven P.M., Nanayakkara P.W.B. (2021). Chest CT in COVID-19 at the ed: validation of the COVID-19 reporting and data system (CO-rads) and CT severity score: a prospective, multicenter, observational study. Chest.

[17] Salvatore C., Roberta F., Angela L., Cesare P., Alfredo C., Giuliano G., Giulio L., Giuliana G., Maria R.G., Paola B.M., Fabrizio U., Roberta G., Beatrice F., Vittorio M. (2021 Jan). Clinical and laboratory data, radiological structured report findings and quantitative evaluation of lung involvement on baseline chest CT in COVID-19 patients to predict prognosis. Radiol. Med..

[18] Zakariaee S.S., Salmanipour H., Naderi N., Kazemi-Arpanahi H., Shanbehzadeh M. (2022 Jul 21). Association of chest CT severity score with mortality of COVID-19 patients: a systematic review and meta-analysis. Clin Transl Imaging.

[19] Hama Amin B.J., Kakamad F.H., Ahmed G.S., Ahmed S.F., Abdulla B.A., Mohammed S.H., Mikael T.M., Salih R.Q., Ali R.K., Salh A.M., Hussein D.A. (2022 May). Post COVID-19 pulmonary fibrosis; a meta-analysis study. Ann Med Surg (Lond).

